# Supplementary material for: The Role of Quarantine on Post-Mortem Performances of Charolaise Young Bulls
Source: Animals (Basel). 2022 Feb 10;12(4):425. doi: 10.3390/ani12040425 (PMC8868343; doi:10.3390/ani12040425)
Supplement: Supplementary file 1 [file animals-12-00425-s001.zip › animals-1581483-supplementary.pdf]

animals

Communication

The Role of Quarantine on Post-Mortem Performances of Charolaise Young Bulls

Alessia Diana [1,†], Matteo Santinello [1], Massimo De Marchi [1,*], Erika Pellattiero [2], and Mauro Penasa [1]

[1] Department of Agronomy, Food, Natural resources, Animals and Environment, University of Padova, 35020 Legnaro, Italy; alessiadiana84@gmail.com (A.D.); matteo.santinello@phd.unipd.it (M.S.); mauro.penasa@unipd.it (M.P.)

[2] Department of Animal Medicine, Production and Health, University of Padova, 35020 Legnaro, Italy; erika.pellattiero@unipd.it

* Correspondence: massimo.demarchi@unipd.it; Tel.: + 39 049 8272627

† Present address: Department of Comparative Pathobiology, Purdue University, West Lafayette, IN 47907, USA

Citation: Diana, A.; Santinello, M.; De Marchi, M.; Pellattiero, E.; Penasa, M. The role of quarantine on post-mortem performances of Charolaise young bulls. *Animals* **2022**, *12*, 425. https://doi.org/10.3390/ani12040425

Academic Editor: Marta López Alonso

Received: 18 January 2022
Accepted: 8 February 2022
Published: 10 February 2022

Publisher's Note: MDPI stays neutral with regard to jurisdictional claims in published maps and institutional affiliations.

Copyright: © 2022 by the authors. Licensee MDPI, Basel, Switzerland. This article is an open access article distributed under the terms and conditions of the Creative Commons Attribution (CC BY) license (http://creativecommons.org/licenses/by/4.0/).

Table S1. Least squares means (LSM) and standard error (SE) of growth and post-mortem performances [1] of Charolaise young bulls (n = 575) for the effect of interaction between farm and quarantine [2].

	Farm 1				Farm 2			
	NO-QUA		QUA		NO-QUA		QUA	
**Trait**	**LSM**	**SE**	**LSM**	**SE**	**LSM**	**SE**	**LSM**	**SE**
ADG_{30} (kg/d)	1.91^{ab}	0.06	2.04^{a}	0.05	1.79^{b}	0.07	2.07^{a}	0.07
$ADG_{31\text{-}end}$ (kg/d)	1.63^{a}	0.02	1.58^{a}	0.02	1.49^{b}	0.03	1.61^{a}	0.03
ADG_{tot} (kg/d)	1.69^{a}	0.02	1.67^{a}	0.02	1.55^{b}	0.03	1.69^{a}	0.03
BW_{final} (kg)	729.17^{a}	4.16	725.55^{a}	4.09	697.54^{b}	5.24	721.82^{a}	5.19
Hot carcass weight (kg)	448.50^{a}	2.75	444.09^{a}	2.67	429.57^{b}	3.46	442.24^{a}	3.40
Dressing percentage (%)	61.49^{a}	0.16	61.25^{a}	0.16	61.65^{a}	0.21	61.35^{a}	0.20
pH_{60}	6.59^{a}	0.02	6.52^{b}	0.02	6.65^{a}	0.02	6.58^{ab}	0.02
SEUROP conformation	5.21^{a}	0.13	4.49^{b}	0.13	4.93^{ab}	0.17	4.66^{ab}	0.17
Fat cover	5.15^{ab}	0.11	4.91^{b}	0.11	5.48^{ac}	0.14	5.89^{c}	0.14

[1] ADG_{30} = average daily gain from day 1 to day 30 of the fattening cycle; $ADG_{31\text{-}end}$ = average daily gain from day 31 to the end of the fattening cycle; ADG_{tot} = average daily gain of the whole fattening cycle; BW_{final} = body weight at the end of the fattening cycle; Hot carcass weight = weight of the carcass after slaughter and after removal of the head, the internal organs, the limbs and the tail; Dressing percentage = the ratio of hot carcass weight to $BW_{final} \times 100$; pH_{60} = pH measured on the *M. gracilis* 60 min post-mortem; SEUROP conformation = development of carcass profiles, and in particular the essential parts (round, back, shoulder) according to the EU Parliament and Council Regulation No 1308/2013 [26]. A number to each letter [and a corresponding lower (–) and upper (+) value, when applicable] was assigned as follows: S = 1, S– = 2, E+ = 3, E = 4, E– = 5, U+ = 6, U = 7, U– = 8, R+ = 9, R = 10, R– = 11, O+ = 12, O = 13, O– = 14, P+ = 15, P = 16, P– = 17; Fat cover = amount of fat on the outside of the carcass and in the thoracic cavity according to the EU Parliament and Council No 1308/2013 [26]. Fat cover was re-classified as follows: 1– = 1, 1 = 2, 1+ = 3, 2– = 4, 2 = 5, 2+ = 6, 3– = 7, 3 = 8, 3+ = 9, 4– = 10, 4 = 11, 4+ = 12, 5– = 13, 5 = 14, 5+ = 15. [2] NO-QUA = animals which followed the standard procedure of the fattening cycle; QUA = animals which followed a 30-day period of quarantine before moving to the building of the standard fattening pens. [a,b,c] Means with different superscript letters within trait are significantly different according to Bonferroni post-hoc adjustment ($p < 0.05$).

Table S2. Least squares means (LSM) and standard error (SE) of growth and post-mortem performances [1] of Charolaise young bulls (n = 575) for the effect of interaction between season of arrival and quarantine [2].

	**Autumn**				**Winter**				**Spring**				**Summer**			
	**NO-QUA**		**QUA**		**NO-QUA**		**QUA**		**NO-QUA**		**QUA**		**NO-QUA**		**QUA**	
**Trait**	**LSM**	**SE**	**LSM**	**SE**	**LSM**	**SE**	**LSM**	**SE**	**LSM**	**SE**	**LSM**	**SE**	**LSM**	**SE**	**LSM**	**SE**
ADG$_{30}$ (kg/d)	1.49^{a}	0.08	1.81^{ab}	0.09	1.69^{ab}	0.13	2.20^{bc}	0.13	2.20^{c}	0.07	2.22^{c}	0.07	2.04^{bc}	0.06	1.98^{bc}	0.06
ADG$_{31\text{-}end}$ (kg/d)	1.56^{ab}	0.03	1.55^{ab}	0.03	1.62^{ab}	0.05	1.65^{ab}	0.06	1.60^{ab}	0.03	1.61^{b}	0.03	1.47^{a}	0.03	1.57^{ab}	0.02
ADG$_{tot}$ (kg/d)	1.56^{a}	0.03	1.59^{ab}	0.03	1.64^{ab}	0.05	1.74^{ab}	0.05	1.71^{b}	0.03	1.73^{b}	0.03	1.57^{a}	0.02	1.65^{ab}	0.02
BW$_{final}$ (kg)	701.34^{b}	6.44	709.29^{ab}	6.53	719.33^{ab}	9.91	734.61^{ab}	10.31	727.58^{ac}	5.26	731.04^{a}	5.39	705.18^{bc}	4.93	719.79^{a}	4.26
Hot carcass weight (kg)	436.12^{ab}	4.29	439.00^{ab}	4.30	440.75^{ab}	6.53	450.54^{ab}	6.66	447.51^{b}	3.46	447.81^{b}	3.53	431.77^{a}	3.25	435.31^{ab}	2.81
Dressing percentage (%)	62.16^{a}	0.26	61.90^{a}	0.26	61.31^{ab}	0.39	61.23^{ab}	0.41	61.60^{a}	0.21	61.48^{a}	0.21	61.22^{ab}	0.19	60.58^{b}	0.17
pH$_{60}$	6.66^{a}	0.03	6.56^{c}	0.03	6.58^{a}	0.04	6.48^{b}	0.04	6.64^{ac}	0.02	6.69^{a}	0.02	6.60^{a}	0.02	6.48^{b}	0.02
SEUROP conformation	4.92^{ab}	0.21	4.51^{ab}	0.21	5.36^{a}	0.32	4.37 ^{ab}	0.33	4.68^{ab}	0.17	4.16^{b}	0.17	5.31^{a}	0.16	5.27^{a}	0.14
Fat cover	5.04^{a}	0.18	5.02^{a}	0.18	5.20^{ab}	0.27	5.57^{ab}	0.28	5.02^{a}	0.14	4.92^{a}	0.15	6.00^{b}	0.13	6.08^{b}	0.12

[1] ADG$_{30}$ = average daily gain from day 1 to day 30 of the fattening cycle; ADG$_{31\text{-}end}$ = average daily gain from day 31 to the end of the fattening cycle; ADG$_{tot}$ = average daily gain of the whole fattening cycle; BW$_{final}$ = body weight at the end of the fattening cycle; Hot carcass weight = weight of the carcass after slaughter and after removal of the head, the internal organs, the limbs and the tail; Dressing percentage = the ratio of hot carcass weight to BW$_{final}$ × 100; pH$_{60}$ = pH measured on the *M. gracilis* 60 min post-mortem; SEUROP conformation = development of carcass profiles, and in particular the essential parts (round, back, shoulder) according to the EU Parliament and Council Regulation No 1308/2013 [26]. A number to each letter [and a corresponding lower (–) and upper (+) value, when applicable] was assigned as follows: S = 1, S– = 2, E+ = 3, E = 4, E– = 5, U+ = 6, U = 7, U– = 8, R+ = 9, R = 10, R– = 11, O+ = 12, O = 13, O– = 14, P+ = 15, P = 16, P– = 17; Fat cover = amount of fat on the outside of the carcass and in the thoracic cavity according to the EU Parliament and Council No 1308/2013 [26]. Fat cover was re-classified as follows: 1– = 1, 1 = 2, 1+ = 3, 2– = 4, 2 = 5, 2+ = 6, 3– = 7, 3 = 8, 3+ = 9, 4– = 10, 4 = 11, 4+ = 12, 5– = 13, 5 = 14, 5+ = 15. [2] NO-QUA = animals which followed the standard procedure of the fattening cycle; QUA = animals which followed a 30-day period of quarantine before moving to the building of the standard fattening pens. [a,b,c] Means with different superscript letters within trait are significantly different according to Bonferroni post-hoc adjustment ($p < 0.05$).
